# Reorganization Tips from a Sarcoma Unit at Time of the COVID-19 Pandemic in Italy: Early Experience from a Regional Referral Oncologic Center

**DOI:** 10.3390/jcm9061868

**Published:** 2020-06-15

**Authors:** Barbara Rossi, Carmine Zoccali, Jacopo Baldi, Alessandra Scotto di Uccio, Roberto Biagini, Assunta De Luca, Maria Grazia Petrongari, Virginia Ferraresi

**Affiliations:** 1 Oncological Orthopedic Unit, IRCCS-Regina Elena National Cancer Institute, 00144 Rome, Italy; carmine.zoccali@ifo.gov.it (C.Z.); jacopo.baldi@ifo.gov.it (J.B.); roberto.biagini@ifo.gov.it (R.B.); 2 Hepato-Biliary and Organ Transplant Unit, School of General Surgery, Sapienza University, 00144 Rome, Italy; allascotto@gmail.com; 3 Department Risk Management Unit, IRCCS-Regina Elena National Cancer Institute, 00144 Rome, Italy; assunta.deluca@ifo.gov.it; 4 Department of Radiation Oncology, IRCCS-Regina Elena National Cancer Institute, 00144 Rome, Italy; mariagrazia.petrongari@ifo.gov.it; 5 Department of Medical Oncology, IRCCS-Regina Elena National Cancer Institute, 00144 Rome, Italy; virginia.ferraresi@ifo.gov.it

**Keywords:** COVID-19, sarcoma, cancer patients, screening, management

## Abstract

Since the World Health Organization declared the novel coronavirus outbreak a global health emergency, Italy’s lockdown was declared on 9 March 2020. Elective orthopedic surgery was forced to stop to allow the healthcare system to face the emergency. However, many orthopedic oncology cases could not be postponed. The aim of this study was to report the experience in managing sarcoma patients and the reorganization of a cancer center in an attempt to maintain it free from COVID-19. A Coronavirus Crisis Unit was established by the health directorate coordination in order to adopt specific procedures. General rules of screening and social distancing were applied in different health settings (entrance check point, hospital inward, outpatient clinic, operative room). Regarding oncologic orthopedics, priority was given to bone and soft tissue sarcomas, metastases and aggressive benign tumors at risk of impending or pathologic fracture. Precise indications were followed to manage first outpatient visits, patients undergoing surgery and follow-up. Meticulous adherence to rules among patients and personnel and collaboration between leadership and medical staff in order to continue to perform multidisciplinary treatment protocols, maintain the availability of infrastructural spaces and source protective equipment, swabs and screening samples have been successful in the aim towards a safe cure for cancer patients.

## 1. Introduction

Since the World Health Organization (WHO) declared the novel coronavirus (SARS-CoV-2) outbreak a global health emergency, Italy’s lockdown was declared on 9 March 2020 [1]. As a result of the lack of early governmental interventions and the undoubted delay in managing the emergency, the Italian health system had to face an ever-increasing number of contagions and patients variably affected by respiratory distress while contrasting the exponential transmission of COVID-19. One month later, according to the activation of the third phase of the emergency announced by the Ministry of Health, many hospitals and medical centers were reorganized in Hub and Spoke Units to ensure the implementation of intensive care beds and new multidisciplinary networks with infectious, medical, pulmonology and resuscitation specialties [2].

Early identification, isolation and referral of selected cases in which hospital care was necessary were mandatory. As learned from the Chinese model, containment measures such as home isolation appear to be the best strategy to break the chain of transmission. The rapid spread of the coronavirus required a complete reorganization of infrastructures which must continue adapting as knowledge of the disease progresses [3,4].

As many other elective specializations, orthopedic surgery was forced to stop at the time of SARS-CoV-2 to allow the national healthcare system to face the emergency [5]. However, similar to trauma injuries, many orthopedic oncology cases could not be postponed [1,5]; these included bone or soft tissues sarcomas, bone metastases and some aggressive benign tumors because of their related risk of impending or pathological fractures. Soft tissue and bone sarcomas are rare malignant tumors, globally accounting for less than 1% of all new cancers diagnoses (<0.2% for bone sarcomas) [6,7]. Management of these patients in reference centers with high levels of expertise and within reference networks and translational clinical trials is the key to improve the prognosis of these rare tumors [6,7,8]. Many patients, from children to elderly people, affected by primary or metastatic malignancies experienced the tragedy of the disease together with the fear of viral contagion. The task of remaining the referral center for “no-COVID-19” oncologic diseases given to our institute by the regional ordinance was challenging as the medical staff was unprepared to face this unprecedented threat together with the well-known frailty of cancer patients. Our institute is located in Rome and it is the reference cancer center for the Lazio region, which is the largest region in central Italy and the second most populated Italian region after Lombardy. According to the 9 April 2020 regional bulletin [9], there were 4429 overall cases: 253 patients died, 644 patients recovered and 3532 were COVID-19-positive patients; of these, 1244 were hospitalized with mild disease, 198 required respiratory support in Intensive Care Units (ICU) and 2090 were in home isolation.

The purpose of this paper is to report the experience in managing oncologic orthopedic activity and how the professional setting was reorganized in order to continue clinical and surgical practice in total safety for healthcare personnel and cancer patients.

## 2. Materials and Methods

### 2.1. Local Setting

The institute’s organization is conceived to enhance clinical assistance, diagnostic and managerial skills throughout the departments that deal with cancer treatment and research [10,11]. The institute does not have an emergency room (ER). The sarcoma unit is a member of the European Reference Network (ERN) for rare tumors “EURACAN” [8] and it also represents a center of excellence for osteoncology.

Although there is limited data on immunosuppressed hosts, cancer is a significant risk factor for infection and development of complications from COVID-19. A higher rate of patients needing mechanical ventilation or ICU admission, as well as a higher mortality rate, is reported for cancer patients in comparison with patients without cancer [12,13,14]. Unfortunately, many medical centers located at the outbreak epicenter, such as Lombardy, could not prevent intra-hospital infections and lacked ICU beds and adequate resources for inpatients [15]. The lower density and the slowdown of viral transmission in central and southern Italy gave more time to our regional healthcare system to reorganize local hospital networks. Despite the potential threat of SARS-CoV-2, to provide proper, prompt and safe treatment to cancer patients was a mainstay of the institute’s governance at the time of the COVID-19 outbreak in Italy. While ensuring all the medical and surgical activities for cancer patients followed international and national guidelines, our institute carefully complied with governmental, ministerial and regional provisions regarding the behaviors to adopt in order to avoid the spread of SARS-CoV-2 [16]. Every medical, diagnostic and surgical specialty adopted dedicated pathways and procedures for its patients according to the single characteristics of care. However, some general rules must be respected to limit the risk of spreading the disease [17].

### 2.2. General Rules

First of all, the medical staff was engaged to contact people, through phone interviews, to ascertain their health and to evaluate any needs. Access to the center for laboratory and/or radio-diagnostic investigations were recommended only in cases of acute need. The visits were booked only online to avoid forbidden waiting lines. Patients were instructed online and by phone to present themselves for visits and exams 30 min before the appointment time to complete the acceptance procedure and to retrieve laboratory and radiological exams only through online reports. Periodic nonessential follow-up visits were canceled or postponed.

Due to the absence of an ER, the second step toward infection and environmental control was to organize a checkpoint at the entrance of the institute where screening of all patients, visitors and medical staff took place before entering the outpatient clinic and hospital. The screening involved measuring body temperature and administering a questionnaire on recent travels, occupation, contact with COVID-19-positive people and/or clusters and symptoms.

Visitors were limited to one care giver at the most, only if necessary, keeping at least one meter of distance from other people. Protective medical masks for patients and caregivers were strongly recommended, as was social distancing and frequent hand washing with hydro-alcoholic gel, which was available in all the transit and/or waiting areas of the institute [13]. A remote consultancy service came into force for those who needed to contact one of the specialists. Chemotherapy, radiotherapy and related follow-up activities continued regularly. A drug domiciliation project for oral therapies was also developed in order to reduce patient hospitalization. Surgical activities proceeded and post-operative checks were regularly delivered. In-ward visits to hospitalized patients were limited to one visitor per day during visiting time, which was reduced to one hour. Visitors were strictly forbidden in the intensive care and resuscitation areas. Patients were instructed to carefully follow the indications provided by the healthcare professionals in all aspects of the center’s activities. Reinforcement of a strict “stay at home when ill” policy was applied both to patients, their families and to medical and paramedic staff in case of traced exposure [18]. Disease management team briefings among multidisciplinary specialists were canceled to avoid crowding and interdepartmental consultations were available through business emails or telecommunication. All staff were instructed that they must wear full personal protective equipment (PPE) and were taught how to don and remove PPE in different settings of care (surgical and medical wards, operative theatre, ambulatory, diagnostic section, etc.) An adequate cleaning and sanitization of spaces was planned if a suspected or confirmed COVID-19-positive patient had been identified.

Within the regional contact-tracing network, the epidemiological survey was always performed in case of COVID-19 positivity detection among both patients and personnel; testing with rhino-pharyngeal swabs in a dedicated service area of both symptomatic and asymptomatic staff members when they came in contact with potential COVID-19 positivity could have been the key to limiting exposures. All personnel with a history of possible COVID-19 contact were constantly monitored by the institute’s occupational doctor.

A coronavirus crisis unit was established by the health directorate coordination in order to adopt all the above-mentioned procedures and to guarantee the diffusion to the personnel through video tutorials and publications on the intranet section of the institute website. All policies, guidelines and creation of ad hoc infrastructural routes helped to create a no-COVID-19 hospital, separating a closed circuit for isolation of suspected COVID-19-positive patients.

### 2.3. Sarcoma Unit Approach

New diagnoses of bone and soft tissues malignant tumors could not be delayed. Surgery for known patients affected by malignant tumors continued to respect the timing of therapeutic schedules. Patients affected by bone metastases or rare benign aggressive tumors at risk of fracture or other adverse events were considered to require attention immediately. Therefore, outpatient waiting lists and surgical interventions needed a new prioritization. In setting priorities, the urgency was assigned for newly diagnosed high- or intermediate-grade sarcomas, relapse detected at follow-up and unstable clinical conditions postoperatively. All activities performed at our institution during the COVID-19 outbreak are summarized in Table 1.

In the same way, the team had to continue the immediate and early postoperative controls and follow-up of patients at high-risk of local recurrence or systemic progression. In all cases, indication for surgical treatment and its most suitable timing were preceded by virtual multidisciplinary tumor board discussions that considered a start with neoadjuvant radiotherapy whenever possible, as defined by current guidelines [6,7]. Preoperative and adjuvant chemotherapy and radiation treatment were planned, giving high priority to malignant bone tumors and high-/intermediate- grade soft tissue tumors. Medical and radiation schedules were regularly performed for patients already undergoing treatment and in the context of a clinical trial whenever patient benefits outweighed the risks and when patient safety was ensured. Radiotherapy was considered urgent for acute spinal cord compression and high priority was given as palliative treatment for bleeding or painful non-pharmacologically responsive inoperable masses or painful bone metastases that were not surgically treated. Whenever possible and considered case by case, hypo fractionated radiation regimens were promoted in order to reduce hospital visits. When feasible, chemotherapy schedules were deferred to reduced clinical visits and blood tests and clinical monitoring of side effects were recommended near homes and under the control of the patient’s general practitioner (GP). Domiciliation of oral therapies was extended to selected patients with metastatic sarcomas. Besides this, both patients and physicians could consult the platform dedicated to telemedicine. To keep the sarcoma unit free of COVID-19, it was essential to control the patients’ access to services. We can distinguish between new cases and known cases.

#### 2.3.1. New Cases

**Figure 1:**
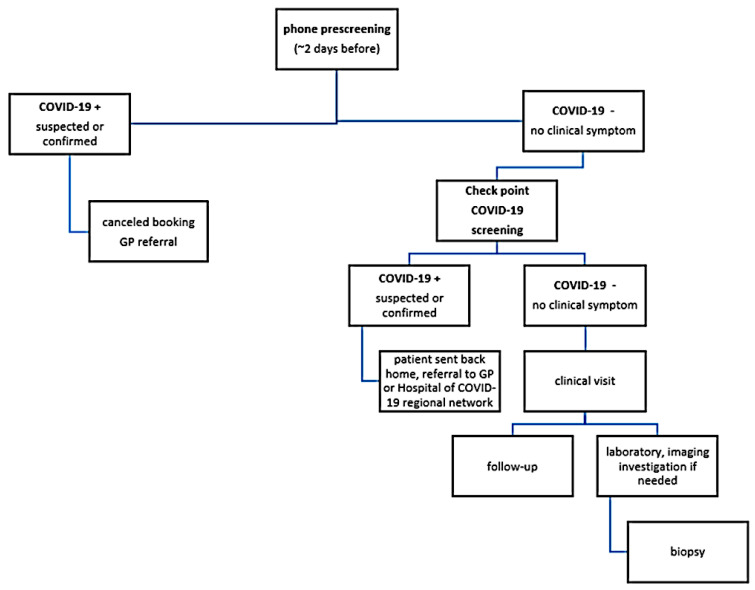
Organizing flow chart for the first outpatient clinic. GP: general practitioner.

In absence of an ER, cancer patients could get access to the orthopedic unit from the outpatient clinic (Figure 1) or from consulting reports from colleagues of other hospitals who guaranteed the urgency.

All patients were pre-screened via phone one or two days before the upcoming visit; these screenings evaluated the patients and their relatives’ symptoms such as cough, shortness of breath, muscle aches, fever and admission to other hospitals within the past 14 days [21]. If any concerning items were reported, they would be furtherly investigated by the GP with a rhino-pharyngeal swab, then monitored at home or referred to the ER. If the consultation was urgent, the on-charge orthopedic doctor could organize the swab in the hospital’s dedicated service area and the visit would be performed the following day if the result was negative.

In case of positive detection of SARS-CoV-2 at the swab, the patient would be addressed to a hospital with an infective disease ward in the Lazio region COVID-19 network if symptoms were consistent or to the GP for home isolation with a bio-containment transportation team. In case of a safe phone prescreening, the attending visit was planned and the patient was instructed to undergo checkpoint screening at the entrance of the institute the day of the orthopedic visit.

After first consultation, latent benign tumors, pseudo-tumor conditions and mid/long-term follow up visits were deviated to teleconsulting or were programmed after resolution of the epidemiologic emergency. Patients with malignant and benign but locally aggressive tumors continued their diagnostic flow-chart leading to active treatment.

#### 2.3.2. Already-Known Patients

Patients already in treatment pathways and follow-up patients (post-operative controls, patients who had to undergo wound dressing, non-surgically treated patients) were included in this group. These patients presented at clinical observation following scheduled protocols. The on-charge doctor assessed medical records and the patient’s priority one week before the outpatient clinic appointment; all nonessential visits were rescheduled or transitioned to telemedicine if possible. Urgent patients affected by sarcomas and/or other malignancies and selected aggressive benign tumors were confirmed as usual protocol [6,7] then called via phone two days before clinical observation for the screening interview following the same flow-chart for new patients.

Patients who had to access the hospital for wound dressing were also considered for risk of dehiscence and infection: when the risk of wound complication was assessed to be low, the patient was addressed to the GP; otherwise, when the risk was considered to be consistent, the patient was invited to the hospital’s wound care outpatient clinic. All patients invited for consultations were triaged at the entrance of the institute for the screening.

#### 2.3.3. Urgent Orthopedic Consultation from Other Hospitals

Oncological orthopedic consultations for patients admitted in other hospitals were only performed after a rhino-pharyngeal swab that was found negative for SARS-CoV-2. The patient and the healthcare professionals were screened at the hospital entrance.

#### 2.3.4. Inter-hospital Inpatient Transfer

Transfers from other hospitals were quite limited and only permitted in selected cases such as when patients were affected by malignant primary tumors or benign aggressive tumors in which the quality of care guaranteed in our specialized hospital could assure a better outcome; patients with bone metastases were treated in general hospitals except in cases where highly demanding surgery was required. A prior orthopedic consultation was mandatory before proceeding to the transfer.

#### 2.3.5. Institute Entrance Check Point Screening

Patients were encouraged to respect social distancing of at least one meter, to wear a medical mask and gloves and to answer a questionnaire interview regarding the risk of contamination. COVID-19 symptoms were investigated again and temperature was checked with a thermal scanner: the referring orthopedic doctor could require a rhino-pharyngeal swab before the visit in any dubious case. Separate infrastructural areas were reserved for suspected COVID-19 patients awaiting confirmation results. For all non-suspected cases, outpatient visits proceeded with fast diagnostic assessment if necessary, and biopsy planning as soon as possible or addressing the patient to a follow up visit based on specific clinical suspect. A person accompanying the patient was admitted to the hospital only if strictly necessary for assistance; he/she also had to be screened and had to respect the same rules applied to the patient.

#### 2.3.6. The Out-Patient Clinic

On patient arrival, the nurse in charge checked that the PPE was correctly worn and assigned a queue number, inviting the patient to find a place in the waiting room. The seats were arranged so as to maintain safe distances in the room, limiting contact and interactions between patients. The patient was allowed to enter the consultation room alone, if possible, and his/her permanence was limited to the time that was strictly necessary. The orthopedic doctor had to wear PPE and limit physical contact as much as possible; the following appointment was postponed after the resolution of the emergency, if possible; telemedicine was encouraged.

#### 2.3.7. Wards and Surgery

Concerning the surgical activities, malignant bone and soft tissue tumors and any pathological fracture or impending fracture were approached following the surgical lists as scheduled in pre-COVID-19 times; patients affected by benign tumors and pseudo-tumors were contacted to advise them that their surgery would be planned after the resolution of the emergency.

**Figure 2:**
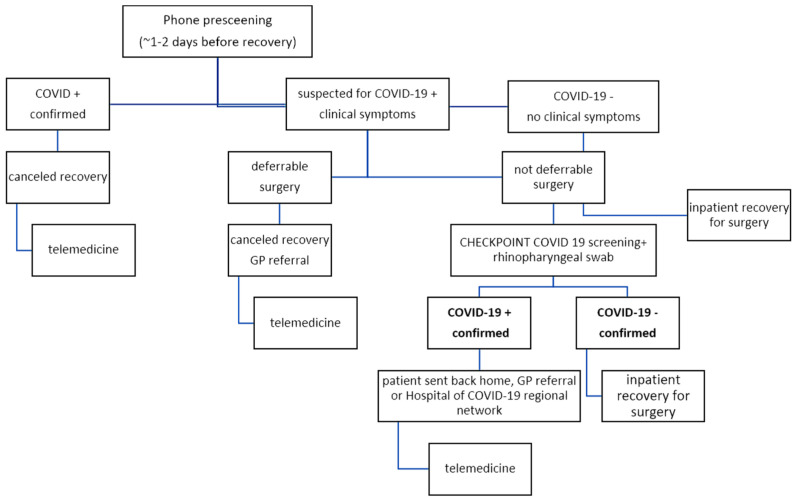
Organizing Flow Chart for inpatient recovery from surgical treatment. GP: general practitioner.

As schematically shown in Figure 2, the on-charge surgeon had to contact the patient two days before access for a telephone prescreening; in absence of risk factors for COVID-19, the patient was invited for inpatient admission; in the presence of one or more risk factors, a rhino-pharyngeal swab was planned and in case of negative results, the inpatient admission would take place the day after. In case of positivity for SARS-CoV-2, the patient was addressed to hospitals of the COVID-19 network of our region if symptoms were consistent or to COVID-19-committed hospital in case urgent surgery was needed, while home isolation and transfer to the GP was recommend if surgery was not urgent and if symptoms were mild or absent. Hospital stay at the time of the COVID-19 outbreak was managed as normal except for social distancing among patients and medical staff when possible and, obviously with all due precautions and PPE. When possible, rooms were reserved for one patient; therapy administration, antibiotic and thrombotic prophylaxis, vital signs assessment, preoperative restaging, preoperative anesthesiology consultation and perioperative care proceeded routinely. Intraoperatively, full PPE including surgical shields and goggles were donned. Surgical times were kept short and operative team personnel were numerically minimized as much as possible, even for more difficult resection and reconstruction procedures. In case of an onset of coughing, dyspnea, fever not related to surgery, anosmia or ageusia during the inpatient stay, the patient was isolated and a rhino-pharyngeal swab was performed. If the swab was positive for infection, the patient would be urgently transferred to a COVID-19-committed hospital. Relatives, care givers and support persons’ access was restricted to one visitor per patient and scheduled for one hour per day. Many bone malignancies such as Osteosarcoma or Ewing sarcoma affect children and our sarcoma unit was conceived to treat young patients aged from 12 to 18 years old; only one parent was admitted during inpatient stay, prior to achieving a written proxy from the other parent for full decision-making autonomy regarding any therapeutic concern, as required by Italian law. Inpatient stay was shortened as much as possible; discharge was mainly planned to be at home, except in selected cases of postoperative rehabilitation which could not be postponed; in these cases, the patient was transferred to a rehabilitation center.

## 3. Results

At the time of the study, after about 40 days of protocol application, the sarcoma unit could apparently be considered no-COVID-19. Seventy-nine patients were hospitalized for surgical treatment from 1 December 2019 to 30 April 2020, 18 more than in the same period of the previous year. Despite the rarity of musculoskeletal tumors, this result can be interpreted as a consequence of centralization of the disease, but also as a successful outcome from the correct adoption of regional inter-hospital and institutional procedures. No inpatient COVID-19 positive cases were found; no positive cases were reported among the unit’s healthcare staff. The selection procedure intercepted a 24-year-old patient with a suspected pelvic chondrosarcoma who should have undergone bone biopsy but had a concomitant interstitial pneumonia; he was addressed to GP consultation due to the clinical suspect of SARS-CoV-2 infection. The oncologic orthopedic consultations from other hospitals were always performed after negative results for COVID-19 from swab screening and this obligation was successful in avoiding the transmission of infection among medical centers. There were no particular perioperative complications for our hospitalized patients; no urgent interventions were canceled because of blood transfusion shortage or a reduction of ICU beds.

## 4. Discussion

COVID-19 has been shown to develop in a wide spectrum of clinical manifestations, from asymptomatic or paucisymptomatic forms, to severe viral pneumonia with respiratory distress up to systemic dysfunctions and death from multiple organ failure. Multiorgan impact of COVID-19 and frailty from comorbidities are strictly connected, as several risk factors including older age, diabetes mellitus, hypertension, immunosuppression and cancer disease are related to the progression of the viral infection [22].

The systemic immunosuppressed state caused by anti-cancer treatments such as chemotherapy or surgery make cancer patients more susceptible to infection from SARS-CoV-2 and this frailty is responsible for a poor prognosis [12]. Despite the rarity of the disease, this statement also applies to bone and soft tissue cancer patients, who are often advanced in age and undergo multimodal treatment. Appointed as a reference center to support cancer patients during this emergency for all the oncologic structures of COVID-19-committed hospitals in the Lazio region, specific guidelines of COVID-19 risk management were developed in our institute in order to maintain the continuity of care with regard to medical treatment and surgery [18,21]. Thus, it was imperative to maintain the hospital’s no-COVID-19 status and the employee and leadership’s aim was to guarantee clean pathways for patients with cancer as much as possible including inpatient hospitals, lab wards, operating rooms and outpatient clinics. In our experience, some principles were considered a priority: first, all cancer patients must be regarded as potentially infected; second, patient access to the sarcoma unit had to be kept untouched from risk of contagion; third, screening needed to be vernacular; and lastly, the orthopedic team had been advised to wear surgical masks and gloves for all patient encounters and to follow strict hand hygiene practices [3,4]. As surgeons, utmost care was given to patients in the preoperative, intraoperative and postoperative settings to minimize the risks of nosocomial spread. Despite worldwide cancellation of elective orthopedic procedures, surgical practice performed for sarcoma patients during the COVID-19 outbreak in our institute was in line with current literary guidance [5,23]. In the same period that Italy dealt with the emergency, the European Society for Medical Oncology (ESMO) emanated recommendations for the management and treatment of patients affected by sarcoma [23]. Our approach was in line with the suggested risk stratification of priorities but some typical surgical cases have to be highlighted: (1) since unresponsive to chemotherapy and radiation, chondrosarcoma was treated with very high priority in the orthopedic unit; (2) bone metastases were treated regardless of the distinction between oligo- or pluri-metastatic patients as the risk for pathological fracture or untreatable pain was not postponed; (3) high priority was given to young growing patients for whom limb salvage surgery required reconstruction with customized implants. Prioritization of urgent cases and accuracy in minimizing risk of COVID-19 infection resulted in optimal tools to preserve our cancer patients.

Already normally dedicated to avant-garde surgery, the emergence of such a crisis provided us with a timely opportunity to evaluate the use of novel technologies in the workplace for our cancer patients. This included the adoption of telemedicine service and tele-rehabilitation initiatives, allowing patients to be reviewed in the comfort of their own homes. A phone help-desk was instituted for video-chat psychological support [23,24]. Videoconferencing tools were adopted to remotely monitor patient outcomes and to educate their care givers [21,25]. The policy of the Institute strongly promoted this virtual interaction, not only to avoid the loss of continuity of care but also to support psychological and emotional stress complained of by patients in such a fragile condition.

We are conscious that many centers in the most infected zones have been unprotected and suddenly overwhelmed by the emergency: our thoughts and daily sacrifices are dedicated to colleagues and patients who directly faced the tragedy of the outbreak [15]. On the contrary, the infection slowdown in regions like ours allowed the preparation of regional governance to reorganize the healthcare network, to commit hospitals for COVID-19-positive patients and to reserve other centers for the treatment of patients that were not infected. Although the fight against the SARS-CoV-2 infection is a priority, cancer centers should make it their mission to do all that is possible to continue to keep their doors open in order to provide valuable care. Emerging literature reiterates the point that our duty is not only to educate but also to provide resources to help patients make decisions regarding treatment during this time of uncertainty. In a period when channeling a large amount of resources for a single patient is in direct conflict with the greater social good, cancer patients especially need individualized and rationed but safe treatments [15,18,21].

## 5. Conclusions

Preservation of cancer patients and security of healthcare professionals have been our mission. We are aware that early coordination of institution-wide efforts has been essential to manage rapidly changing information. Collaboration between the leadership and medical staff, reorganization among interdepartmental units in order to continue to perform multidisciplinary treatment protocols, the availability of infrastructural spaces, PPE, swabs and screening samples have been our winning weapons. Despite the unpredictability of the future and the resources available, our hope is that our proposal of what has been a successful strategy until now could be helpful to any other oncologic center while fighting this war.

## Figures and Tables

**Table 1:** Main surgical indications in treatment of bone and soft tissues tumors considered urgent during COVID-19 outbreak.

Biopsy for undetermined nodule/mass or osteolysis with risk of cancer diagnosis; repetition of biopsy in case of previous discordant diagnosis likely to be malignant
Resection of biopsy-proven malignant tumor or recurrence with risk of disease progression and/or metastasis
Resection of tumor followed by 3D-printed or custom-made endoprosthetic reconstruction
Fixation of impending or actual pathological fractures in patients with life expectancy > 3 months [19]
Spinal cord decompression for spinal column tumor (in case of weakness, bowel/bladder dysfunction, sensory changes, pain; radiographic evidence of cord compression; intractable pain)
Postoperative complications requiring surgery [20] (i.e., periprosthetic fracture or prosthetic loosening, infection-related complications, compressive hematoma, disease progression)
